# Altered cytokine profiles and elevated lactate levels in children with autism spectrum disorder: indications of a dysregulated immune-metabolic network?

**DOI:** 10.3389/fpsyt.2026.1904470

**Published:** 2026-10-12

**Authors:** Nicole Beyer, Theresia Volk, Hella Luksch, Melanie Ring, Nora C. Vetter, Angela Roesen-Wolff, Min Ae Lee-Kirsch, Veit Roessner

**Affiliations:** 1 Department of Child and Adolescent Psychiatry and Psychotherapy, Faculty of Medicine, Technische Universität Dresden, Dresden, Germany; 2 Department of Pediatrics, Faculty of Medicine, Technische Universtität Dresden, Dresden, Germany; 3 German Centre for Child and Adolescent Health (DZKJ), Partner Site Leipzig/Dresden, Dresden, Germany; 4 Department Psychologie, Fakultät Naturwissenschaften, Medical School Berlin (MSB), Berlin, Germany

**Keywords:** autism spectrum disorder, cytokines, IL-1β, immune-metabolic network, immune system, lactate, mitochondrial dysfunction, TNF-α

## Abstract

**Introduction:**

Autism spectrum disorder (ASD) is a lifelong neurodevelopmental condition characterized by difficulties in social communication and restricted, repetitive behavior. The etiology and pathophysiology of ASD remain largely unknown; however, a complex interplay of genetic and environmental factors is widely assumed. More recently, altered cytokine/chemokine profiles and a potential vulnerability to mitochondrial alterations have been proposed.

**Methods:**

We measured blood lactate and pyruvate and quantified a cytokine/chemokine panel including IL-1β, IL-2, IL-6, TNF-α, IL-8, IFN-β, RANTES, MCP-1/CCL2, MIP-1α/CCL3, MIP-1β/CCL4, CCL11/Eotaxin-1 and CXCL10/IP-10 in a group of autistic children in comparison to a non-autistic age-, gender- and IQ-matched control group.

**Results:**

We observed elevated lactate levels, lower concentrations of IL-1β and TNF-α in autistic- compared with control children. In addition, lactate, MIP-1β/CCL4 and TNF-α was significantly associated with behavioral measures of autistic behavior. Pyruvate, the lactate/pyruvate ratio and all other analytes did not differ between groups.

**Discussion:**

We conclude that the observed cytokine/chemokine profile may reflect a dysregulated immunological network in autistic children. Elevated lactate may point to metabolic abnormalities with possible mitochondrial involvement. However, unchanged pyruvate and lactate/pyruvate ratio do not support overt mitochondrial dysfunction. These findings require replication in larger studies.

## Introduction

Autism spectrum disorder (ASD) is a lifelong neurodevelopmental condition characterized by difficulties in social communication and restricted, repetitive behavior. In this manuscript, we use the term “autistic children” to refer to children with ASD and to individuals with subclinical autistic traits. The etiology and pathophysiology of ASD remain incompletely understood; however, a complex interplay of genetic susceptibility and environmental factors is widely assumed. In recent years, two lines of research have gained particular attention: altered peripheral immune signaling, especially cytokine and chemokine profiles, and metabolic abnormalities that may indicate mitochondrial involvement (1–3).

The biological relevance of immune signaling for ASD is plausible because the immune system plays an essential role in brain development and synaptic plasticity. Immune cells such as microglia are involved in synaptic pruning, and altered immune activation may affect neural circuits relevant for behavior, cognition, and social functioning (4). In addition, reduced synaptic density may contribute to reorganization and formation of specialized neural networks (5). Because synaptic density reaches a peak in early childhood and is followed by major phases of synapse elimination during early and mid-adolescence (6), altered immune activation during these sensitive developmental windows could have particularly adverse effects on neurodevelopment and the manifestation of autistic symptoms. Furthermore, cytokines can cross the blood-brain barrier and influence brain function by disrupting neuroplasticity, synaptic functioning, and the regulation of behavior, and may thus also contribute to autistic symptoms (7).

Cytokines, chemokines, and interferons are key mediators of immune signaling. Various cohort studies have reported associations between atypically regulated neonatal cytokine profiles and an increased likelihood of later receiving an ASD diagnosis (8, 9). Moreover, peripheral inflammatory alterations have repeatedly been discussed as possible contributors to atypical neurodevelopment and autistic behavior (10, 11). In addition to deviant neonatal cytokine profiles that may increase the likelihood of a later ASD diagnosis, higher concentrations of cytokines have also been associated with increased autistic symptoms in older children, adolescents and adults (12–15).

However, findings on specific cytokines in ASD remain inconsistent. Although altered concentrations of TNF-α, IL-1β, IL-6, IL-8, and CCL11/Eotaxin-1 have repeatedly been reported, results differ across age groups, study designs, and analytic strategies (14, 16–19). In a recent meta-analysis, we addressed this heterogeneity by conducting a sensitivity analysis that controlled for potential biases, including medication use, small sample sizes, missing control groups, and diagnostic procedures not based on ICD-10 or DSM-5 criteria (20). This analysis indicated that observed effects for TNF-α, IL1-ß, IL-6, and IL-8 were not age-dependent. At the same time, significantly elevated concentrations in autistic compared with control groups were retained only for IL-1ß, IL-4, IL-8, and IFN-γ, whereas IL-6 and TNF-α no longer differed significantly between groups. This heterogeneity suggests that immune alterations in ASD are unlikely to be captured by single markers alone and may instead reflect a broader but variable inflammatory profile. With regard to interferons, IFN-γ has been examined most frequently and has provided initial indications of how immune processes may influence social behavior (21). In contrast to IFN-γ, IFN-β exerts mainly antiviral and inhibitory effects and has been described as immunomodulatory in the brain (22). Because IFN-β may reduce proinflammatory cytokines such as IL-1β and TNF-α, decreased IFN-β levels could contribute to increased inflammatory activity in ASD. In addition, several studies on altered cytokine profiles in autistic compared with control groups have also examined chemokines such as MCP-1/CCL2, CCL11/Eotaxin-1, RANTES, MIP-1α/CCL3, MIP-1β/CCL4, and CXCL10/IP-10 (12, 13, 18). Reported alterations in these markers suggest that dysregulation of proinflammatory chemokines may contribute to the pathophysiology of social impairments and ASD.

A second relevant line of research concerns metabolic dysfunction and possible mitochondrial involvement in ASD. Mitochondrial alterations may be linked to oxidative stress and immune dysregulation, suggesting a potential interplay between metabolic and inflammatory mechanisms (23, 24). However, it remains unclear whether mitochondrial dysfunction in ASD is a cause, a consequence, or an associated feature (25). Several biomarkers commonly used to screen for mitochondrial dysfunction, including lactate, pyruvate, alanine, and creatine kinase. Previous work has linked ASD to elevated lactate, pyruvate, and lactate/pyruvate ratios, although findings are not uniform (23). Elevated serum lactate has long been discussed as a possible indicator of a mitochondrial subtype in ASD (26). More recently, increased cerebral lactate values were reported in autistic adults in a magnetic resonance spectroscopy (MRS) study, further supporting the relevance of altered energy metabolism in at least a subgroup of affected individuals (27).

Against this background, the present study examined peripheral immune markers together with metabolic markers in autistic children compared with non-autistic controls matched for age, sex, and IQ. Specifically, we asked whether.

- serum lactate, pyruvate, and the lactate/pyruvate ratio differ between groups;
- serum concentrations of selected cytokines and chemokines (IL-1β, IL-2, IL-6, TNF-α, IL-8, IFN-β, RANTES, MCP-1/CCL2, MIP-1α/CCL3, MIP-1β/CCL4, CCL11/Eotaxin-1, and CXCL10/IP-10) differ between groups; and
- these biomarkers are associated with dimensional measures of autistic behavior.

In addition, since several factors are known to influence circulating cytokine and lactate levels, including body mass index (BMI (28), dietary habits (29), physical activity (30), sleep quality (31), recent stress and socioeconomic status (32), we analyzed whether groups differ with respect to these factors.

## Materials and methods

### Participants

Participants were recruited at the Department of Child and Adolescent Psychiatry and Psychotherapy and at the Department of Pediatrics, both of the University Hospital Carl Gustav Carus in Dresden, Germany. Participants were primarily male (83.6%) and to a lesser extent female (16.4%) and in the age range between six and fourteen years (*M* = 10.71 ± 1.96).

The data were collected between January 2019 and early 2023. The autistic group consisted of children recruited from the Autism Centre at the University Hospital Carl Gustav Carus in Dresden, Germany. Children received a clinical diagnosis of autism spectrum disorder (ICD-10: F84.0, F84.1 or F84.5) (33) based on gold-standard diagnostic assessment including the Diagnostic Observation Schedule – Version 2 (ADOS-2; 34) and the Diagnostic Parent Interview for Autism – Revised (ADI-R; 35) before participation in this study. The sample presented here is part of a greater study investigating whether nutritional supplementation with CoQ10 improves wellbeing in autistic children and adolescents (Jahn et al, unpublished manuscript). Because of the planned CoQ10 supplementation, the following exclusion criteria were defined: treatment with thyroxine, soy allergy, and initiation of a new therapy during the same time as the study period (existing therapies could be continued). With regard to the planned serum cytokine analyses, additional exclusion criteria were autoimmune or autoinflammatory diseases, acute inflammatory reactions or infections, current medication use.

Before participation, the non-autistic children underwent a diagnostic interview conducted by trained staff to rule out autism spectrum disorder. Individuals with psychiatric or neurological disorders, with the exception of adjustment disorder (F43.2), or somatic diseases affecting the immune or central nervous system were excluded. All participants and their parents gave written informed consent. Participants received financial compensation for their time, and travel expenses were reimbursed. The study was approved by the Ethics Committee of the Technische Universität Dresden (reference number: EK 427102017) and was conducted in accordance with the Declaration of Helsinki.

Parents completed the following questionnaires: the Social Communication Questionnaire (FSK, (36)), the Social Responsiveness Scale (SRS, (37)), the Affective Reactivity Index-Parent Version (ARI-P, (38)), the Marburger Assessment Scale for Asperger’s Syndrome (MBAS, (39)) and the Child Behavior Checklist (CBCL, (40)). For the present study, data from the SRS, FSK, ARI-P and MBAS are reported because they offer information on autistic traits and related behavioral characteristics.

The groups did not differ in sex (*χ*^2^(2) = 5.74, *p* = .057), chronological age (*t*(53) = 1.84, *p* = .20, Cohen`s *d* = .24, 95% CI [-0.32, 0.81]), and IQ (*t*(46.296) = 6.03, *p* = .42, Cohen`s *d* = .21, 95% CI [-0.77, 0.36]). Please refer to **Table 1** for further information on the clinical sample characteristics of the autistic group.

**Table 1:** Clinical characteristics *of* the autistic group.

Continuous variables	*N*	*M*	*SD*
IQ	43	97.02	20.63
ADOS-2			
Social Affect	43	9.14	4.43
Restrictive and Repetitive Behaviors	43	1.88	2.25
Total Score	43	10.88	5.09
Comparison Score	43	6.07	2.48
ADI-R			
Social Interaction	41	15.61	4.92
Communication	41	11.68	3.68
Restrictive and Repetitive Behaviors	41	3.73	2.05
Categorical variables		*%*	
ICD-10 ASD Diagnoses			
F84.0	16	37.2%	
F84.1	11	25.6%	
F84.5	16	37.2%	

ADOS-2, Autism Diagnostic Observation Schedule, Second Edition; ADI-R, Autism Diagnostic Interview– Revised; ICD-10, International Statistical Classification of Diseases and Related Health Problems – Tenth Revision.

### Cytokine analysis

Peripheral blood was collected from each participant, and serum was isolated and analyzed for cytokine quantification. We applied Cytometric Bead Array (CBA) Kits from BD Biosciences (San Jose, USA).

### Lactate and pyruvate analysis

For lactate and pyruvate analysis, peripheral blood was collected using a citrate Monovette and frozen at -80 °C. Subsequent analyses were carried out by an external laboratory (Biovis).

### Statistical analysis

As the data were predominantly non-normally distributed, group differences were examined using Mann-Whitney U tests (see **Table 2**). Subsequently, variables that differed significantly between groups, together with age and sex, were entered into a binary logistic regression model to examine their association with group status (autistic vs. non-autistic). Finally, bivariate correlation analyses were conducted for the variables that differed between groups and the total scores of the following behavioral measures: FSK (36), SRS (37), ARI-P (38) and the MBAS (39). Moreover, since dietary habits, BMI, socioeconomic status and stress are known to influence cytokines we analyzed dietary habits, BMI, socioeconomic status and parent rated stress (and added data to **Supplementary Tables S1** and **S2**).

**Table 2:** Cytokine profile for the control and the autistic group.

Variable	Median (IQR)	U	p	r	group	n	Mean (± SD)
pyruvate	102 (80.00 - 132.00)	316.5	.387	0.11	control group	18	194.47 (± 414.01)
					autistic group	41	137.86 ( ± 200.06)
Lactate	1670 (1500- 1970)	199.00	.005	0.36	control group	18	1453.71 (± 452.78)
					autistic group	41	1815.84 ( ± 479.51)
lactat/pyruvate ratio	17 (15.00 - 19.40)	290.00	.193	0.17	control group	18	16.43 (± 2.88)
					autistic group	41	18.29 ( ± 4.30)
IL-1β	19.79 (8.5-42.97)	224.5	.013	0.32	control group	18	48.20 (± 45.19)
					autistic group	42	24.46 ( ± 26.70)
CXCL10/IP-10	36.45 (14.3 - 50.77)	270.00	.145	0.19	control group	17	29.28 (± 18.62)
					autistic group	42	45.49 ( ± 39.14)
CCL11/EOTAXIN-1	58.89 (42.18 - 85.39)	292.00	.165	0.18	control group	18	58.83 (± 28.88)
					autistic group	42	70.63 ( ± 37.22)
IL-6	4.75 (0.0 - 13.97)	305.00	.578	0.07	control group	16	17.31 (± 37.20)
					autistic group	42	11.00 ( ± 17.08)
MCP-1/CCL2	557.76 (389.87 - 826.72)	342.00	.561	0.07	control group	18	663.26 (± 355.61)
					autistic group	42	640.86 ( ± 391.51)
IL-2	24.88 (9.03 - 75.04)	269.5	.643	0.06	control group	14	71.52 (± 108.32)
					autistic group	42	77.87 ( ± 131.39)
MIP-1α/CCL3	97.48 (48.33 - 142.54)	240.00	.050	0.25	control group	17	132.79 (± 71.22)
					autistic group	42	114.19 ( ± 128.85)
IL-8	33.2 (20.82 - 58.15)	288.5	.149	0.19	control group	18	33.39 (± 19.23)
					autistic group	42	59.03 ( ± 71.07)
IFN-β	138.29 (40.40 - 306.27)	234.00	.439	0.10	control group	13	263.46 (± 332.49)
					autistic group	42	419.30 ( ± 1431.04)
**TNF-α**	15.56 (4.26 - 54.24)	98.00	<.001	0.52	control group	15	124.72 (± 144.66)
					autistic group	42	23.90 ( ± 39.44)
MIP-1β/CCL4	58.09 (25.98 - 110.24)	147.00	.001	0.43	control group	16	135.56 (± 95.01)
					autistic group	42	60.27 ( ± 65.30)
RANTES	12359.2 (6656.30 - 25693.95)	352.00	.933	0.01	control group	17	17174.86 (± 15367.84)
					autistic group	42	17255.04 ( ± 14128.01)

Bold dotted variables symbolize a significant difference between groups.

## Results

Mann-Whitney-U Tests revealed differences between groups for lactate (*U* = 199.0, *p* = .005, *r* = .36), IL-1β (*U* = 224.5, *p* = .013, *r* = .32), TNFα (*U* = 98.0, *p<*.001, *r* = .52) and MIP-1β/CCL4 (*U* = 147.0, *p* = .001, *r* = .43) (see **Table 2** for details). However, correcting for multiple testing to avoid type I error (i.e. Bonferroni correction, indicating that the significance level was adjusted to the number of tests (*p* = .005/15)) revealed that solely TNFα (*U* = 98.0, *p<*.001, *r* = .52) and MIP-1β/CCL4 (*U* = 147.0, *p* = .001, *r* = .43) remained significant.

### Logistic regression analysis

A binary logistic regression model using the Enter method was computed to examine whether lactate and cytokines that differed between groups (IL-1β, TNF-α, MIP-1β/CCL4), together with age and sex, were associated with group status (0 = non-autistic, 1 = autistic, see **Table 3**). The overall model was significant, *χ*²(6) = 26.04, *p* <.001, indicating better fit than the null model. The model explained between 37.2% (Cox & Snell R²) and 54.1% (Nagelkerke R²) of the variance in the dependent variable. The logistic regression model thus demonstrated good discriminative ability, with an area under the receiver operating characteristic curve (ROC-AUC) of 0.906. At a classification threshold of 0.50, the model correctly classified 92.7% of the autistic group (sensitivity) and 46.7% of the control group (specificity), resulting in an overall classification accuracy of 80.4%. The Hosmer–Lemeshow test did not indicate a significant lack of model fit, χ²(7) = 7.20, *p* = .408. Thus, although specificity was relatively low at the 0.50 classification threshold, the model demonstrated good overall discrimination and no evidence of significant lack of fit in the present sample.

**Table 3:** Log, regression of lactate, cytokines, age, gender and group (non-autistic, autistic).

	Regression coefficient	Standard error	Wald	df	Sig.	Exp(B)	Lower value	Upper value
**Lactate**	.002	.001	4.317	1	.038	1.002	1.000	1.004
**TNF-α**	-.059	.025	5.454	1	.020	.943	.898	.991
MIP-1β/CCL4	.020	.014	2.091	1	.148	1.020	.993	1.049
IL-1β	.022	.026	.736	1	.391	1.022	.972	1.076
age	-.102	.240	.182	1	.670	.930	.564	1.444
**gender**	-2.327	1.129	4.246	1	.039	.098	.011	.893
constant	-.134	2.826	.002	1	.962	.875		

Bold dotted variables symbolize a significant difference between groups.

Higher TNF-α levels were associated with lower odds of belonging to the autistic group (OR = .943 [.898,.991], *p* = .020), whereas higher lactate levels were associated with higher odds of belonging to the autistic group (OR = 1.002 [1.000, 1.004]), *p* = .038). In addition, female sex was associated with lower odds of belonging to the autistic group compared with male sex (OR = .098 [.011,.893], p =.011). Both, IL-1β (OR = 1.022 [.972, 1.076]), *p* = .391) and MIP-1β/CCL4 (OR = 1.020 [.993, 1.049]), *p* = .148) did not emerge as significant predictors in the multivariable model.

### Correlation analysis between lactate, TNF-α, IL1β, MIP-1β/CCL4 and autistic behavior

We conducted bivariate correlation analyses between lactate, cytokines (TNF-α, IL-1β, MIP-1β/CCL4) and the respective total score of MBAS, SRS, FSK and ARI-P. In order to avoid α inflation and to control for multiple testing, we adjusted the significance level from *p* = .05 to *p* = .005 (p= .05/9 tests). We observed significant correlations for MIP-1β/CCL4 x FSK total score (*r* = -.372, *p* = .005), TNF-α x SRS total score (*r* = -.487, *p <*.001), TNF-α x FSK total score (*r* = -.473, *p<*.001) and lactate x SRS total score (*r* = .457, *p* <.001) and lactate x FSK total score (*r* = .417, *p=*.001), all other *p* >.006.

## Discussion

The present study identified a specific peripheral immune-metabolic pattern in autistic children. Compared with non-autistic controls, autistic children showed higher lactate concentrations and lower TNF-α, IL-1β, and MIP-1β/CCL4 concentrations, whereas pyruvate, the lactate/pyruvate ratio, and the remaining analytes did not differ between groups. Moreover, lactate and TNF-α remained informative in the multivariable model, and lactate, TNF-α, and MIP-1β/CCL4 were associated with dimensional behavioral measures. Taken together, these findings are more consistent with a coordinated biological profile than with an isolated single-marker effect. However, the reduced levels of TNF-α and IL-1β observed in autistic children do not align with the typical trend, also seen in our current meta-analysis (20). This meta-analysis (98 studies involving 4,236 autistic individuals and 3,333 non-autistic individuals) found elevated levels of IL-1β and TNF-α overall. However, the analysis included individuals of all age groups, and a large proportion of the studies exhibited a high risk of bias. This significantly diminishes the interpretive value of individual studies and highlights the heterogeneity of the data and the disease. It also emphasizes the influence of age and developmental stage, as well as the presence or absence of medication history, on immune response. Finally, it is highly likely that several immune phenotypes co-occur with ASD subtypes. This complicates the interpretation of findings observed in relatively small groups of participants. It can be assumed that a subgroup of autistic children exhibits altered regulation of the innate immune response, leading to a reduced pro-inflammatory response under specific conditions. Thus, although meta-analyses support the presence of altered peripheral cytokine profiles in ASD, they also demonstrate significant variability between studies in the direction and magnitude of the effects (14, 16, 20). Likewise, studies of plasma cytokines and chemokines have linked altered immune markers to behavioral impairment in autistic children (12, 13). Against this background, our data do not support a simple model of globally increased peripheral inflammation. Rather, they suggest selective dysregulation within an immune network, in which reduced TNF-α, IL-1β, and MIP-1β/CCL4 may characterize a specific biological state in a subgroup of autistic children.

In this context, IL-1β appears repeatedly implicated across the broader ASD immune literature (15), whereas chemokine findings, including MIP-1β/CCL4, have been more variable across individual reports and therefore particularly warrant replication.

TNF-α deserves particular attention because it participates not only in inflammatory signaling but also in neuroimmune communication and developmental processes. In this context, lower peripheral TNF-α concentrations may reflect altered proinflammatory baseline activity, impaired neuroimmune communication, or compensatory immune regulation rather than straightforward immune weakness. This interpretation is compatible with experimental work showing that TNF-α can negatively regulate type 1 immune activation (41) and with ASD data reporting lower TNF-α expression together with greater early social impairment (42). The concurrent reduction of TNF-α, IL-1β, and MIP-1β/CCL4 in the present study therefore further argues for a network-level interpretation instead of treating each analyte as an independent signal.

The co-occurrence of this cytokine/chemokine pattern with elevated lactate also suggests possible immune-metabolic coupling. In recent immunometabolism research, lactate is no longer viewed merely as a by-product of glycolysis but as a signaling metabolite that can modulate macrophage, dendritic-cell, and T-cell function and reshape inflammatory responses (43–45). Although the present data cannot establish mechanism, they support the working hypothesis that elevated lactate may be linked to a potentially compensatory or reprogrammed peripheral immune milieu in a subgroup of autistic children.

The behavioral correlations add an important dimensional perspective. Lactate was positively associated with SRS and FSK total scores, whereas TNF-α and MIP-1β/CCL4 showed negative associations with these measures. This pattern suggests that the identified markers may relate not only to categorical group membership but also to the severity of social-communicative and related behavioral characteristics. At the same time, the multivariable model indicates that not all altered analytes act as independent predictors; rather, IL-1β and MIP-1β/CCL4 may represent supportive components of a broader immune-metabolic profile. This interpretation is in line with prior work suggesting that altered cytokine profiles may be linked to symptom severity and may help delineate biologically meaningful subgroups, with sex potentially modifying some of these associations (12, 13, 15). One plausible hypothesis for future studies is therefore that a behaviorally more affected subgroup may also show a more pronounced peripheral immune-metabolic signature.

With respect to metabolism, the lactate finding is best interpreted cautiously. Elevated lactate is compatible with altered peripheral energy metabolism or metabolic stress, but the unchanged pyruvate concentrations and unchanged lactate/pyruvate ratio do not support overt mitochondrial dysfunction. This distinction is important because the ASD-mitochondria literature is itself heterogeneous and increasingly suggests that mitochondrial abnormalities may be especially relevant for subgroups rather than for ASD as a whole (2, 23, 25, 46). In addition, increased cerebral lactate has recently been reported in autistic adults by magnetic resonance spectroscopy, again supporting the possibility of metabolically affected subgroups (27). Accordingly, our data fit better with a model of metabolic involvement, or at most possible mitochondrial participation, than with a claim of manifest mitochondrial disease.

Because acute inflammatory reactions and infections were excluded, the observed biomarker constellation may reflect basal peripheral physiology rather than an acute inflammatory state. However, the cross-sectional design does not allow us to determine whether this pattern represents a stable trait or a state-dependent signature influenced by sleep, stress, diet, or other contextual factors (see also **Supplementary Tables S1** and **S2**). It is also conceivable that immune-metabolic markers in ASD are developmentally dynamic. Neonatal studies have reported altered cytokine and chemokine profiles long before diagnosis, suggesting that the direction and salience of peripheral markers may vary across developmental windows (18, 19). This developmental perspective may also help explain why peripheral IL-6 was not altered here despite experimental literature implicating IL-6-mediated pathways during maternal immune activation in prenatal neurodevelopmental risk (47). Biological sex should likewise be considered as a potential moderator, both because sex was significant in our model and because sex shapes immune responses across the lifespan (48). Future studies should therefore test whether the present profile is stable within individuals, varies by developmental stage or sex, and characterizes a behaviorally more affected subgroup within the autism spectrum.

The absence of group differences in IL-2, IL-6, IFN-β, RANTES, MCP-1/CCL2, MIP-1α/CCL3, CCL11/Eotaxin-1, and CXCL10/IP-10 should also be interpreted carefully. In a modest cross-sectional sample, null findings do not imply that these markers are biologically unimportant. Rather, they may reflect selective involvement, limited statistical power, developmental specificity, or context-dependent immune variation. In this vein it should be noted that the Mann–Whitney U tests yielded small-to-large observed effect sizes (r = 0.11 – 0.52). Sensitivity analyses (80%) based on the available sample sizes indicated that the study was initially powered to detect relatively large effects. Furthermore, given that the effect size *r* is typically modest in magnitude, the attainment of a non-significant result does not inherently imply unexpectedness. Thus, due to the modest size of the control group, the study may not have possessed sufficient sensitivity to reliably detect minor effects. However, the effects observed for TNF-α as well as MIP-1β/CCL4 are of particular significance, given that they obtain significant differences with comparatively high effect size despite the relatively small size of the control group. This interpretation is compatible with studies that likewise found no clear group differences for some peripheral cytokine panels but still reported relationships with quantitative traits or clinical subgroups (49). Overall, the present findings support a hypothesis-generating model in which a subgroup of autistic children shows a selective, potentially compensatory peripheral immune-metabolic shift characterized by elevated lactate and reduced TNF-α, IL-1β, and MIP-1β/CCL4 (but see diminishing effects of lactate and IL-1β if data were Bonferroni corrected). Future studies should move beyond single-marker approaches and test small candidate panels, ideally combining lactate, TNF-α, IL-1β, and MIP-1β/CCL4 with dimensional behavioral measures in larger, longitudinal, and sex-sensitive samples. In addition, studies that use urine as an additional peripheral sample, might also be promising, noninvasive studies to consider, when the metabolic status and possible deviations of autistic children should be analyzed. Thus, metabolomic studies in autistic children have identified alterations in urinary amino acids, tryptophan and purine metabolism, oxidative stress, mitochondrial pathways, neurotransmitter-related metabolites, and microbiota-derived metabolites (see 50, 51). More recent untargeted metabolomic studies provide further support for the potential of urinary metabolites as candidate biomarkers and suggest that urinary metabolic profiles may capture biologically relevant ASD sub-phenotypes. However, the majority of extant urine studies are cross-sectional in nature, thus necessitating longitudinal studies to ascertain temporal stability, predictive value, and clinical utility of these biomarkers. Longitudinal investigations further suggest that immune and metabolic alterations in ASD may vary across developmental stages and biological compartments. For instance, Pardo and colleagues (1) longitudinally assessed immune mediators in serum and cerebrospinal fluid and found limited correspondence between peripheral and central immune profiles, emphasizing that peripheral biomarkers may not fully reflect neuroimmune processes. More recently, longitudinal multi-omics analyses of umbilical cord blood and childhood serum have identified metabolic and immune-related molecular alterations that may precede and persist after the emergence of an ASD diagnosis. In conjunction with large-scale peripheral metabolomic studies, these findings provide support for a systems-level and longitudinal approach that integrates peripheral metabolic, immune, and, where feasible, CNS-related measures to identify biologically meaningful subgroups of ASD and potential therapeutic targets. Moreover, multimarker and data-driven subgrouping approaches, potentially including machine-learning-assisted clustering, may be particularly useful for determining whether the observed pattern reflects a stable endophenotype, a state-dependent signature, or a biologically meaningful subtype within the autism spectrum. In addition, multicenter studies with larger and more balanced cohorts are recommended, since our findings with a small, mono-centered rather male cohort is limited with respect to generalizability.

## Conclusion

In summary, the present study points to a selective peripheral immune-metabolic shift in autistic children, characterized by elevated lactate and lower TNF-α, IL-1β, and MIP-1β/CCL4 concentrations. Rather than indicating a uniform proinflammatory state, this pattern is more consistent with a selective and potentially compensatory alteration within a broader immune-metabolic network. The associations with dimensional behavioral measures further suggest that these markers may be related to symptom burden and subgroup structure, not only to categorical group status. At the same time, unchanged pyruvate concentrations and an unchanged lactate/pyruvate ratio argue against overt mitochondrial dysfunction. Given the small cross-sectional sample, the present findings should therefore be interpreted as hypothesis-generating rather than definitive. In addition, receiver operating characteristic (ROC) analysis, sensitivity, specificity, and predictive performance of combined biomarkers would substantially enhance the translational significance of the findings – the absence may be interpreted as limitation of the study. In the same vein we acknowledge that an internal validation procedure such as bootstrapping or cross-validation would provide a stronger assessment of the stability of our applied statistical model. Replication in larger, longitudinal, sex-sensitive cohorts is now required to determine whether a multimarker profile centered on lactate, TNF-α, IL-1β, and MIP-1β/CCL4 identifies a stable endophenotype, a state-dependent signature, or a biologically meaningful subgroup within the autism spectrum.

## Data Availability

The raw data supporting the conclusions of this article will be made available by the authors, without undue reservation.
