# Supplementary material for: Altered cytokine profiles and elevated lactate levels in children with autism spectrum disorder: indications of a dysregulated immune-metabolic network?
Source: Front Psychiatry. 2026 Oct 12;17:1904470. doi: 10.3389/fpsyt.2026.1904470 (PMC13697830; doi:10.3389/fpsyt.2026.1904470)
Supplement: Supplementary file 1 [file Table1.docx]

Table S1 Descriptive Statistics and Independent-Samples t Tests Comparing the Autism and Control Groups

| **Variable** | **Autism, *M* (*SD*)** | **Control, *M* (*SD*)** | ***t*** | ***df*** | ***p*** | | **Mean Difference** | **95% CI** |
| --- | --- | --- | --- | --- | --- | --- | --- | --- |
| BMI | 0.2914 (0.07909) | 0.2720 (0.06956) | 0.86 | 26.24 | .399 | 0.01935 | | [−0.02706, 0.06576] |
| Winkler Index, Total | 14.069 (4.4875) | 12.250 (4.8097) | 1.24 | 29.25 | .224 | 1.8190 | | [−1.1720, 4.8099] |
| CSHQ: Bedtime Resistance | 7.6000 (2.64035) | 6.8000 (0.94112) | 1.11 | 17.50 | .284 | 0.80000 | | [−0.72365, 2.32365] |
| CSHQ: Sleep Onset Delay | 1.9333 (0.96115) | 1.4667 (0.63994) | 1.57 | 24.37 | .130 | 0.46667 | | [−0.14817, 1.08150] |
| CSHQ: Sleep Duration | 4.8667 (1.64172) | 4.1333 (1.24595) | 1.38 | 26.11 | .180 | 0.73333 | | [−0.36028, 1.82694] |
| CSHQ: Sleep Anxiety | 6.4667 (2.44560) | 5.2000 (1.26491) | 1.78 | 20.99 | .089 | 1.26667 | | [−0.21180, 2.74513] |
| CSHQ: Night Wakings | 3.6667 (0.81650) | 3.6000 (0.98561) | 0.20 | 27.06 | .842 | 0.06667 | | [−0.61131, 0.74465] |
| CSHQ: Parasomnias | 9.4000 (2.38447) | 8.8667 (2.41622) | 0.61 | 28.00 | .548 | 0.53333 | | [−1.26211, 2.32878] |
| CSHQ: Sleep-Disordered Breathing | 3.3333 (0.61721) | 3.4667 (1.30201) | −0.36 | 19.99 | .724 | −0.13333 | | [−0.90942, 0.64275] |
| CSHQ: Daytime Sleepiness | 13.7333 (3.12745) | 13.2000 (3.14416) | 0.47 | 28.00 | .645 | 0.53333 | | [−1.81217, 2.87884] |
| CSHQ: Total Sleep Disturbance Score | 48.1333 (8.23639) | 44.3333 (6.57557) | 1.40 | 26.69 | .174 | 3.80000 | | [−1.78653, 9.38653] |
| PSSP: Total Score | 23.4146 (6.97487) | 15.0000 (6.41872) | 4.34 | 29.65 | < .001 | 8.41463 | | [4.45173, 12.37754] |

*Note.* *M* = mean; *SD* = standard deviation; *t* = independent-samples *t* statistic; *df* = degrees of freedom; *p* = two-tailed significance level; CI = confidence interval; CSHQ = Children's Sleep Habits Questionnaire; PSSP = [instrument abbreviation as used in the original dataset]. The mean difference was calculated as Autism − Control. Welch's *t* test (equal variances not assumed) is reported. *N* varied across measures because of missing data.
