# Supplementary material for: Altered cytokine profiles and elevated lactate levels in children with autism spectrum disorder: indications of a dysregulated immune-metabolic network?
Source: Front Psychiatry. 2026 Oct 12;17:1904470. doi: 10.3389/fpsyt.2026.1904470 (PMC13697830; doi:10.3389/fpsyt.2026.1904470)
Supplement: Supplementary file 2 [file Table2.docx]

**Table S2 - *Descriptive Statistics and Independent-Samples t Tests of Dietary Characteristics by Group***

| **Dietary characteristic** | **Autism, *M* (*SD*)** | **Control, *M* (*SD*)** | ***t*** | ***df*** | ***p*** | **Mean Difference** | **95% CI** |
| --- | --- | --- | --- | --- | --- | --- | --- |
| High-carbohydrate diet | 4.85 (1.12) | 4.56 (0.89) | 1.01 | 34.65 | .320 | 0.29 | [−0.29, 0.87] |
| High-protein diet | 3.95 (0.96) | 4.07 (0.80) | −0.46 | 30.08 | .652 | −0.12 | [−0.64, 0.41] |
| High-fat diet | 3.85 (0.98) | 4.20 (0.86) | −1.29 | 28.33 | .207 | −0.35 | [−0.90, 0.20] |
| Eats all foods | 3.28 (1.69) | 4.00 (1.71) | −1.44 | 27.43 | .162 | −0.73 | [−1.76, 0.31] |
| Composition/meal times vary considerably | 2.20 (1.22) | 2.94 (1.24) | −2.02 | 27.43 | .053 | −0.74 | [−1.49, 0.01] |
| Balanced diet | 3.30 (1.57) | 3.63 (1.36) | −0.77 | 31.82 | .446 | −0.33 | [−1.18, 0.53] |
| Savory diet | 3.33 (1.14) | 3.75 (1.13) | −1.27 | 28.05 | .214 | −0.43 | [−1.11, 0.26] |
| Eats meat/fish daily | 4.05 (1.15) | 4.07 (1.28) | −0.04 | 23.06 | .965 | −0.02 | [−0.80, 0.76] |

*Note.* *M* = mean; *SD* = standard deviation; *t* = independent-samples *t* statistic; *df* = degrees of freedom; *p* = two-tailed significance level; CI = confidence interval. Mean differences were calculated as Autism − Control. Welch's *t* test (equal variances not assumed) is reported. *N* varied slightly across variables because of missing data. Data were collected via Likert scale (1-6, i.e. 1 means low-carbohydrate diet to 6 high-carbohydrate diet)
